# Biliary tract visualization using near-infrared imaging with indocyanine green during laparoscopic cholecystectomy: results of a systematic review

**DOI:** 10.1007/s00464-016-5318-7

**Published:** 2016-11-14

**Authors:** S. L. Vlek, D. A. van Dam, S. M. Rubinstein, E. S. M. de Lange-de Klerk, L. J. Schoonmade, J. B. Tuynman, W. J. H. J. Meijerink, M. Ankersmit

**Affiliations:** 1 Department of Surgery, VU University Medical Centre, De Boelelaan 1118, 1081 HV Amsterdam, The Netherlands; 2 Epidemiology and Biostatistics, VU University, Amsterdam, The Netherlands; 3 Medical Library, VU University, Amsterdam, The Netherlands

**Keywords:** Near-infrared imaging, ICG, Laparoscopic cholecystectomy, Biliary tract visualization, Intraoperative cholangiography

## Abstract

**Background:**

Near-infrared imaging with indocyanine green (ICG) has been extensively investigated during laparoscopic cholecystectomy (LC). However, methods vary between studies, especially regarding patient selection, dosage and timing. The aim of this systematic review was to evaluate the potential of the near-infrared imaging technique with ICG to identify biliary structures during LC.

**Methods:**

A comprehensive systematic literature search was performed. Prospective trials examining the use of ICG during LC were included. Primary outcome was biliary tract visualization. Risk of bias was assessed using ROBINS-I. Secondly, a meta-analysis was performed comparing ICG to intraoperative cholangiography (IOC) for identification of biliary structures. GRADE was used to assess the quality of the evidence.

**Results:**

Nineteen studies were included. Based upon the pooled data from 13 studies, cystic duct (Lusch et al. in J Endourol 28:261–266, 2014) visualization was 86.5% (95% CI 71.2–96.6%) prior to dissection of Calot’s triangle with a 2.5-mg dosage of ICG and 96.5% (95% CI 93.9–98.4%) after dissection. The results were not appreciably different when the dosage was based upon bodyweight. There is moderate quality evidence that the CD is more frequently visualized using ICG than IOC (RR 1.16; 95% CI 1.00–1.35); however, this difference was not statistically significant.

**Conclusion:**

This systematic review provides equal results for biliary tract visualization with near-infrared imaging with ICG during LC compared to IOC. Near-infrared imaging with ICG has the potential to replace IOC for biliary mapping. However, methods of near-infrared imaging with ICG vary. Future research is necessary for optimization and standardization of the near-infrared ICG technique.

**Electronic supplementary material:**

The online version of this article (doi:10.1007/s00464-016-5318-7) contains supplementary material, which is available to authorized users.

Laparoscopic cholecystectomy (LC) is one of the most frequently performed surgical procedures worldwide [2, 3]. The most serious complication is bile duct injury with an incidence 0.3–1.5% [4–6]. Bile duct injury has significant impact on quality of life and survival. Since the introduction of LC, prevention of complications has been a priority of surgeons [7]. To avoid complications such as bile duct injury, instruments have been developed to minimize risk. One of the strategies is the critical view of safety (CVS) during the dissection of the gallbladder according to Strasberg [8].

Despite introduction of CVS, the use of modern high-resolution cameras and angled optics, the incidence of bile duct injury still remains 0.42% [9]. Most common causes of bile duct injury are poor identification of the biliary tract and inflammatory changes such as acute cholecystitis. To overcome risk of bile duct injury, extra intraoperative visualization techniques, such as intraoperative ultrasound and cholangiogram (IOC), have been introduced [10].

IOC is a technique that aids the surgeon to identify the biliary anatomy, possible common bile duct stones and anatomical variations during LC. The use of IOC is debateable. Advocates of the technique argue IOC should be used routinely during LC in order to avoid bile duct injury. Adversaries argue that the technique is not practically feasible, because additional trained staff is required during surgery [5]. Furthermore, incision of the cystic duct is required which could lead to possible bile duct injury. Also, IOC provides additional harmful radiation for the patient [5, 11, 12]. Another disadvantage is that partial dissection of Calot’s triangle should be performed before IOC can be utilized and, therefore, bile duct injury can occur before IOC.

Recently, intraoperative visualization of the bile ducts using near-infrared light and the fluorescent dye indocyanine green (ICG) were introduced during cholecystectomy [13]. This technique provides real-time imaging of the biliary tract even before dissection of Calot’s triangle.

ICG is an intravenously delivered agent, which when stimulated by near-infrared light (700–900 nm) provides fluorescent visualization of vascular and biliary structures. ICG is a water-soluble dye with peak spectral absorption at 800 nm. After intravenous injection, ICG is bound to plasma proteins and, therefore, remains intravascular. ICG is metabolized almost exclusively by hepatic parenchymal cells and secreted into the bile. Peak concentration in the bile occurs between 30 min and 2 h after injection, whereas peak concentration in the arterial system is reached within 1–2 min [14].

Near-infrared imaging with ICG during LC has only recently been introduced [15, 16]. Under near-infrared light, the biliary structures are fluorescently highlighted, potentially aiding anatomical identification and prevention of bile duct injury. Benefits of ICG include that it is less invasive and no incision of the cystic duct is required, nor is the patient exposed to radiation. Furthermore, ICG has the potential to identify the biliary tract before dissection of Calot’s triangle, whereas IOC is generally performed after dissection of the CD.

Currently, near-infrared imaging with ICG is rapidly expanding to other surgical areas. The use of near-infrared imaging with ICG for LC is suggested to be safe and feasible [17–19]. However, between the published studies there is wide variation in technical and procedure-related factors, such as dosage, timing of ICG administration and patient pathology.

Primary question of this systematic review is to evaluate how the near-infrared imaging technique with ICG is used during LC in order to identify biliary structures. Secondary questions include: what is the influence of dosage and timing on biliary tract visualization before and after dissection of Calot’s triangle? What is the influence of obesity and gallbladder disease aetiology on biliary tract visualization? And does near-infrared imaging with ICG provide more frequent biliary tract visualization compared to IOC?

## Methods

This systematic review was conducted in accordance with the preferred reporting items for systematic reviews and meta-analysis statement [20]. No approval of the medical ethics committee was required.

### Criteria for considering studies for this review

#### Eligibility criteria

Articles were included if they fulfilled the following criteria: (1) the study describes the use of near-infrared imaging with ICG; (2) reports on laparoscopic cholecystectomies in humans for treatment of gallbladder disease (e.g. cholecystolithiasis and cholecystitis); and (3) used a prospective design (i.e. cohort study or randomized controlled trial). To avoid overlapping patient data in duplicate publications, the article with the largest sample size was included. No publication date or publication status restrictions were imposed. Only studies published in English, German or Dutch were included.

#### Types of outcome measures

Primary outcome of this review was extrahepatic biliary tract visualization of the cystic duct [1], common bile duct (CBD) and common hepatic duct (CHD) before and after dissection of Calot’s triangle. Secondary outcomes were comparison of ICG to IOC, dosage and timing of administration of ICG and body mass index (BMI).

### Search methods for selection of studies

#### Electronic searches

A comprehensive search was performed in the bibliographic databases PubMed, Embase.com, the Cochrane Library (via Wiley) and Web of Science from inception of the databases to the 8 February 2016, in collaboration with a medical librarian (LS). Search terms included controlled terms (Mesh in PubMed, Emtree in Embase) as well as free text terms. Free text terms were only used in the Cochrane Library and Web of Science. The following terms were used (including synonyms and closely related words) as index terms: ‘biliary tract surgical procedures’ or ‘biliary tract’ or ‘biliary tract diseases’ and ‘cholangiography’ or ‘spectroscopy’ or ‘near-infrared’ or ‘surgery, computer-assisted’ and ‘indocyanine green’ or ‘fluorescent dyes’. The full search strategies for all the databases are given in "Supplementary Information S1".

#### Searching other sources

Systematic reviews and narrative review articles that were identified during the search were checked for additional references.

### Data collection and analysis

#### Selection of studies

**Fig. 1:**
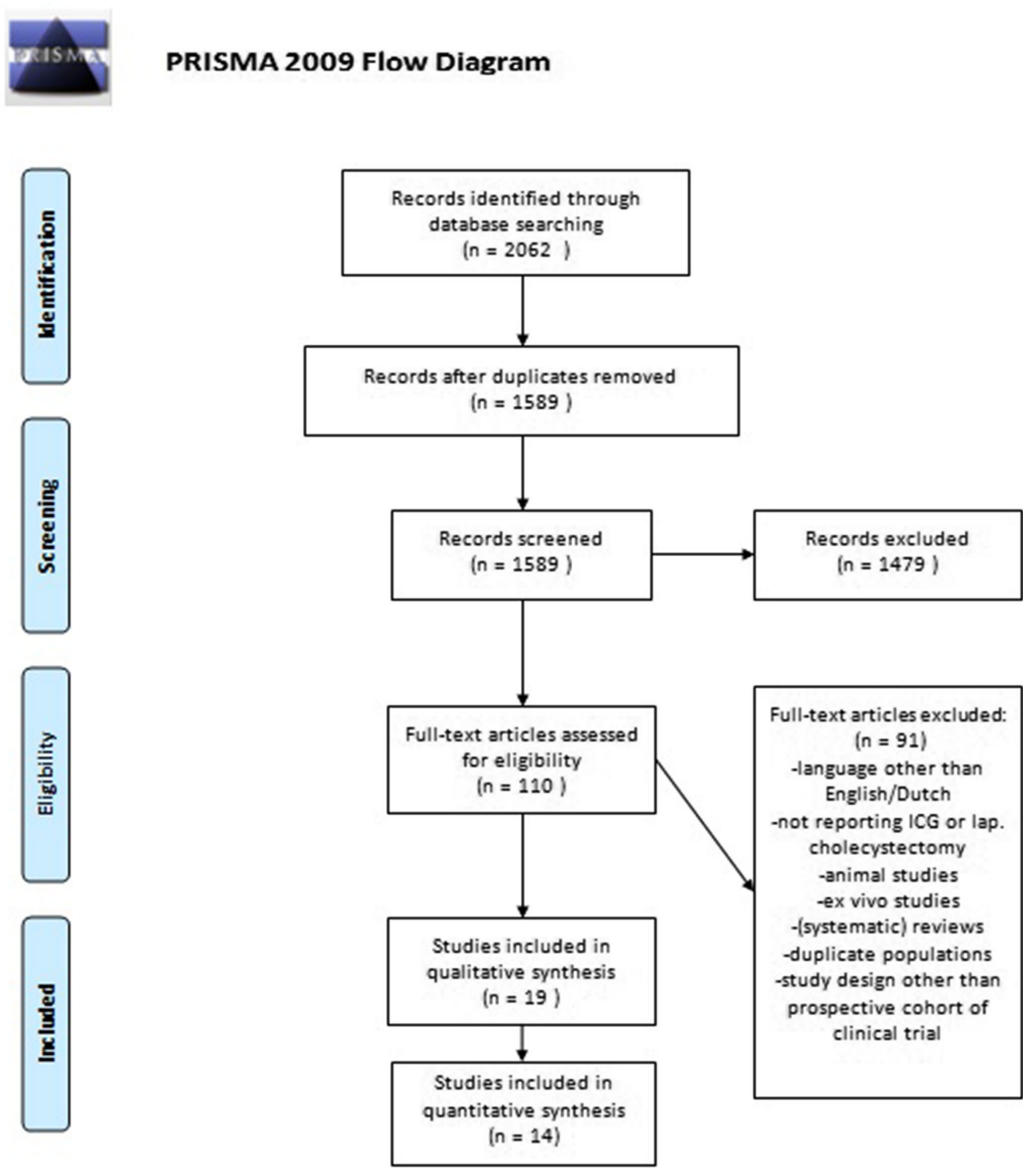
PRISMA—flowchart of search strategy (8 February 2016)

Article selection was performed by three authors independent of one another (DvD, SV and MA). Titles and abstracts were first screened, and when unclear, the full text of an article was examined. In case of disagreement regarding inclusion or exclusion, a paper was discussed to establish consensus. To avoid overlapping patient data in duplicate publications, the article with the largest sample size was included. The process of inclusion is shown in a flow chart (Fig. 1).

#### Data extraction and management

Data were extracted by three authors (SV, DvD and MA) independent of one another using a data extraction form that included information on: publication details; dosage and timing of ICG administration; camera system used; general patient characteristics; general surgical procedure characteristics, including conversion and complication rate and operating time; LC indications and information on biliary tract visualization.

#### Risk of bias assessment

Two authors (SV and MA) assessed the risk of bias for studies that reported comparisons between ICG and IOC using the Cochrane’s ROBINS-I tool (Risk Of Bias In Non-randomized Studies of Interventions) [21]. The following items were assessed: bias due to confounding; selection bias; bias in measurement of interventions; bias due to missing data; bias in measurement of outcomes; and bias in the selection of the reported result. Each domain was scored as low, moderate, serious or critical risk of bias. Overall risk of bias of the included study was scored serious if serious risk of bias was scored in at least one domain. If no serious risk of bias was scored in any domain, the study would receive an overall moderate risk of bias.

#### Measures of treatment effect

The primary outcome is dichotomous (i.e. identification biliary structure). Prevalence of biliary duct visualization for the CD, CBD, CHD and the cystic duct junction (CD-j) is given as proportions. Weighted mean biliary duct visualization was calculated through pooled analysis in a random effects model with 95% confidence intervals using MedCalc software (Oostende, Belgium).

ICG visualization was compared to IOC for the CD, CBD and CHD. Outcomes are weighted by inverse variance in a random effects model. Treatment effect is presented as relative risk with 95% confidence intervals. These analyses were conducted in RevMan5 [22]. Heterogeneity between studies was assessed through a number of manners: Eye-Ball, *Q* test and the *I* ^2^ statistic. Technical failure of IOC was not excluded from calculations, but analysed as not able to visualize the bile duct structures.

#### Data synthesis

The overall quality of the evidence and strength of recommendations was evaluated using GRADE [23]. At the start of GRADE assessment process, we assumed high quality for all studies and we downgraded the quality of the evidence for each comparison by one to two levels depending on the seriousness of the violations in each domain.

To assess the risk of bias for a comparison, the risk of bias tables for each study in that comparison was considered. For each comparison, the risk of bias was considered serious (−1) if a majority of the evidence in the studies included in the meta-analysis (in terms of number of participants) scored serious risk of bias. For consistency, we considered a majority of the following four items: an *I* ^2^ value of more than 50%, statistical significance of heterogeneity (*p* < 0.05), large variation in size effect and no overlapping of confidence intervals for downgrading (−1). If half of the items were scored for inconsistency, the variation in size effect and confidence intervals were considered decisive. For imprecision of results, serious imprecision leading to downgrading (−1) was judged if a comparison included fewer than 22,000 participants or if wide confidence intervals around the effect estimate were reported. Indirectness of the evidence was not an issue in this review, because the population, intervention, comparison and outcomes were directly correlated with the review question. Summary of Findings tables were generated for the primary analyses and for the primary outcome measures only. The quality of the evidence is described as:

- High: further research is very unlikely to change our confidence in the estimate of effect;
- Moderate: further research is likely to have an important impact on our confidence in the estimate of effect and may change the estimate;
- Low: further research is very likely to have an important impact on our confidence in the estimate of effect and is likely to change the estimate.
- Very low: the true effect is likely to be substantially different from the estimate of effect.

A ‘Summary of Findings’ table was created using GRADEpro software [24].

## Results

An overview of included studies is provided in Table 1. In total, 19 studies were included for qualitative evaluation. Of 19 studies [15, 16, 25–41], one non-randomized controlled trial was identified [27] and 18 prospective cohort studies. In total, 772 patients were reported, with a 0.5% (1/197 patients) conversion rate and 1.7% (10/578 patients) complication rate.

**Table 1:** Study characteristics

References	Type of study	Visualization techniques (s)	Patients	F:M		BMI (range) [SD]	Age (range) [SD]	Included indication	Conversion	Complication	Camera system	OT (range) [SD]
Aoki [15]	PCH	NIR-ICG, CI	14	6	8	–	61 (43–72)	SCL	–	0	HP	–
Ishizawa [16]	PCH	NIR-ICG, CI	52	23	29	23.2 (18.2–32.9)	59 (28–78)	SCL	–	0	HP	142 (91–366)
Ishizawa [31]	PCH	NIR-ICG, CI, SI	7	6	1	20.4 (16.6–27.4)	–	SCL	–	–	HP	–
Kaneko [32]	PCH	NIR-ICG, CI, SI (9)	28	18	10	20 (18–42)	51 (22–78)	SCL	0	0	HP	151 (98–343)
Buchs [26]	PCH	NIR-ICG, CI, SI, Rbt	12	8	4	28.5 (20–39)	47 (31–69)	SCL	0	0	DVI	85 (57–125)
Buchs [27]	NRCT	NIR-ICG vs. CI SI, Rbt	44	31	13	27 [4.1]	48 [12]	SCL	0	3/23 (ICG) 1/21 (control)	DVI	85 [22]
Dip [30]	PCH	NIR-ICG, CI	65	35	30	20 (16–40)	42 (19–73)	SCL	–	–	KSE	120 (30–80)
Schols [37]	PCH	NIR-ICG, CI	30	19	11	26.7 (19.7–36.8)	53 (26–81)	SCL	1	0	KSE	–
Spinoglio [38]	PCH	NIR-ICG, CI, SI, Rbt	45	33	12	24.7 (19–43)	48 (23–76)	SCL	0	1	DVI	67 (35–110)
Tagaya [39]	PCH	NIR-ICG, CI, SI (4)	15	9	6	(19.5–27.3)	54 (37–69)	SCL	0	0	Os/HP	88 (68–118)
Boni [25]	PCH	NIR-ICG, CI	52	31	21	–	–	17 SCL 35 ACC	–	0	KSE	54 [13]
Dip [29]	PCH	NIR-ICG, CI	45	24	21	28.4 [6.5]	49 [14.76]	28 SCL 17 ACC	–	0	KSE	66 [19]
Osayi [35]	PCH	NIR-ICG, CI	82	64	18	31.5 [8.2]	43 [14]	SCL	–	0	SE	78 [30]
Prevot [36]	PCH	NIR-ICG, CI	23	21	2	–	45 (18–81)	SCL	0	2	KSE	72 (40–200)
Verbeek [41]	PCH	NIR-ICG, CI	14	–	–	25 (19–40)	61 (26–76)	SCL	–	–	mF	–
Larsen [34]	PCH	NIR-ICG, CI	35	26	9	28 (19–38)	48 (18–74)	33 SCL 2 ACC	–	0	Os	43 (22–135)
Van Dam [40]	PCH	NIR-ICG, CI	30	21	9	27.5 [4.3]	50 [17]	SCL	–	3	Os	71 [20]
Kono [33]	PCH	NIR-ICG, CI	108	49	59	23.5 (15.6–42.2)	56 (19–92)	102 SCL 6 ACC	–	–	HP	–
Dip [28]	PCH	NIR-ICG, CI	71	42	29	53% > 30 47% < 30	–	53 SCL 18 ACC	–	0	KSE	–

*PCH* prospective cohort, *RCH* retrospective cohort, *NRCT* non-randomized controlled trial, *NIR* near infrared, *ICG* indocyanine green, *CI* conventional image, *SI* single incision, *Rbt* Robot, *F:M* female versus male ratio, *BMI* body mass index (kg/m^2^) with [SD] or (range); Age, in years with [SD] or (range), *SCL* symptomatic biliary disease, *ACC* acute cholecystitis, *HP* Hamamatsu photonics, *DVI* Da Vinci System/Intuitive, *KSE* Karl Storz Endoskope, *Os* Olympus, *mF* mini-Flare, *SE* Stryker Endoscopy

Of 19 studies, 13 reported near-infrared imaging with ICG during laparoscopic cholecystectomy [15, 16, 25, 28–30, 33–37, 40, 41] and six studies reported multiple laparoscopic techniques, including single incision and robotic surgery [26, 27, 31, 32, 38, 39].

Average time of first ICG administration varied between 74 min prior to surgery up to after tracheal intubation. Most studies administered ICG between 45 and 60 min prior to surgery. Timing of a second administration was reported on different moments during the surgical procedure in order to identify the cystic artery (CA). The CA was identified in 85.9% [SD 8.33] of the cases [32, 34, 37]. For near-infrared visualization, the use of six different camera systems has been reported.

In total, 14 studies included patients with only uncomplicated gallbladder disease (cholecystolithiasis, chronic cholecystitis or gallbladder polyp), encompassing 311 patients [15, 16, 26, 27, 30–32, 35, 37–41], and five studies included both complicated and uncomplicated gallbladder disease (acute cholecystitis, biliary pancreatitis, cholestasis and post-ERCP), encompassing 461 patients [25, 28, 29, 33, 34]. In total, 78 of 461 patients suffered complicated gallbladder disease.

### Biliary tract visualization per dosage of ICG

Several dosage schemes were used: a fixed dosage of 2.5 mg ICG was used in nine studies [16, 26, 27, 31, 33, 35, 37–39], a dosage of 0.05 mg/kg bodyweight was used in six studies [28–30, 32, 34, 40], a dosage of 0.5 mg/kg bodyweight was used in two studies [25, 36] and two studies reported other dosage schemes [15, 41]. For analysis of biliary tract identification, the studies providing information on dosage scheme were stratified by fixed dosage (2.5 mg) and dosage per kilogram bodyweight (0.05 mg/kg) as given in Table 2.

**Table 2:** Biliary tract visualization per dosage scheme

Study	*n*	Adm. timing (mins)	CD before	CD after	CBD before	CBD after	CHD before	CHD after
Biliary tract visualization with a 2.5-mg fixed dosage of ICG *n* (%)								
Ishazawa [16]	52	30	52 (100)	52 (100)	–	–	50 (96.2)	52 (100)
Ishazawa [31]	7	15^a^	5 (71.4)	–	–	–	7 (100)	–
Buchs [26]	12	45	11 (91.7)	12 (100)	6 (50)	10 (83.3)	4 (33.3)	8 (66.7)
Schols [37]	30	15^a^	29 (96.7)	–	25 (83.3)	–	–	–
Spinoglio [38]	45	45	42 (93.3)	44 (97.8)	41 (91.1)	44 (97.8)	40 (88.9)	44 (97.8)
Osayi [35]	82	74	46 (56.1)	78 (95.1)	31 (37.8)	63 (76.8)	29 (35.4)	57 (69.5)
Kono [33]	108	–	88 (81.5)	103 (95.4)	–		94 (87.0)	100 (92.6)
Weighted mean % (95% CI)			86.5 (71.2–96.6)	96.5 (93.9–98.4)	67.3 (35.5–92.1)	86.6 (67.1–98.0)	76.8 (51.2–94.7)	88.9 (73.5–98.2)
Biliary tract visualization with a 0.05-mg per kg bodyweight dosage of ICG *n* (%)								
Kaneko [32]	28	15	26 (92.8)	–	–	–	27 (96.4)	–
Dip [30]	65	60	50 (76.9)	65 (100)	50 (76.9)	65 (100)	–	–
Dip [29]	45	60	44 (97.8)^b^	–	36 (80.0)^b^	–	27 (60.0)^b^	–
Larsen [34]	35	15^a^	–	–	–	–	–	–
van Dam [40]	30	15^a^	10 (33.3)	29 (96.7)	20 (66.7)	26 (86.7)	–	–
Dip [28]	71	60	71 (100)	–	62 (87.3)	–	50 (70.4)	–
Weighted mean % (95% CI)			85.2 (60.2–98.9)	98.4 (92.4–99.9)	78.7 (70.3–86.0)	95.3 (73.4–99.0)	76.6 (54.5–92.9)	

Visualization per dosage group. Number of biliary structure identifications *n* (% proportion) before and after dissection of Calot’s triangle. Timing of ICG administration is equal (average 37.3 and 37.5 min before surgery) for both groups

*ICG* indocyanine green, *timing* timing of administration, *mins* minutes, *CD* cystic duct, *CBD* common bile duct, *CHD* common hepatic duct

^a^ICG administration after anaesthesia

^b^Biliary structure identification before and during dissection

Seven studies were stratified to the fixed dosage group encompassing 336 patients [16, 26, 31, 33, 35, 37, 38]. Six studies were stratified to the 0.05 mg/kg bodyweight group encompassing 274 patients [28–30, 32, 34, 40]. One study reported on visualization of the CD-j before dissection with positive identification in 35 of 35 cases [34]. No studies reported on the CHD and CD-j visualization after dissection. Two studies have used a dosage scheme of 0.5 mg/kg bodyweight, of which one has evaluated visualization of the biliary structures before and after dissection in 23 patients [36]. Two included studies with different dosage schemes [15, 41] did not report on biliary tract visualization.

In addition to the CD, CBD, CHD, other structures or aberrant anatomy were identified in 70/772 (9.1%) of the patients. Mostly, the confluence of the right and left hepatic duct was visualized [16, 30, 34]. Other identified structures include: an extra CA [40], additional hepatic duct or aberrant course of the CD [16, 30, 34, 40] and a CBD cyst [30].

### ICG versus IOC visualization

**Fig. 2:**
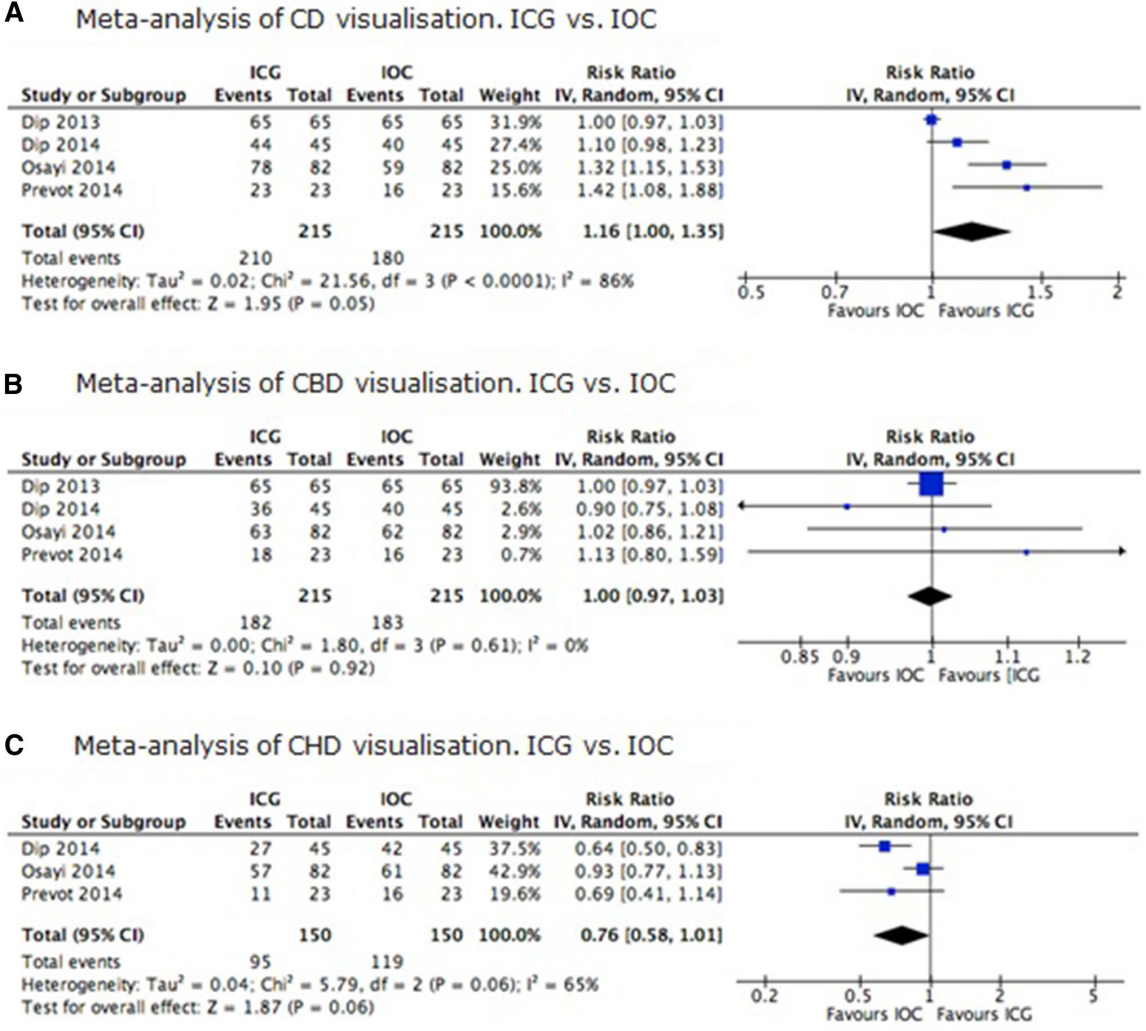
**A** Meta-analysis of CD visualization. ICG versus IOC. **B** Meta-analysis of CBD visualization. ICG versus IOC. **C** Meta-analysis of CHD visualization. ICG versus IOC

In total, four studies compared the use of ICG with IOC in 215 patients [29, 30, 35, 36]. Risk of bias was scored moderate for visualization of the CD and CBD and serious for visualization of the CHD (Table 3). Results of biliary structure visualization with ICG and IOC are presented in Fig. 2A–C. There is moderate quality evidence that visualization of the cystic duct with ICG is better than using the IOC (RR 1.16; 95% CI 1.00–1.35), and, respectively, moderate and low quality evidence for the visualization of the CBD (RR 1.00; 95% CI 0.97–1.03) and CHD (RR 0.76; 95% CI 0.58–1.01)with ICG compared to visualization with IOC. None of the estimated effect sizes were statistically significant (Table 4). All three comparisons were downgraded for serious imprecision because few participants were examined. The comparison of the CHD also scored serious risk of bias due to possible selection bias.

**Table 3:** Risk of bias, ROBINS-I

ROBINS-I	Dip [30]	Dip [46]	Osayi [35]	Prevot [36]
Participants	65	45	82	23
Domain				
Bias due to confounding	Low	Low	Low	Low
Bias in selection of participants into the study	Low	Low	Serious	Serious
Bias in measurement of interventions	Low	Low	Low	Low
Bias due to departures from intended interventions	Low	Low	Low	Low
Bias due to missing data	Low	Low	Low	Low
Bias in measurement of outcomes	Moderate	Moderate	Moderate	Moderate
Bias in selection of the reported result	Low	Low	Low	Low
Overall	Moderate	Moderate	Serious	Serious

**Table 4:** GRADE summary of evidence

*ICG compared to IOC for identification of the biliary ducts* Patient or population: identification of the biliary ducts Setting: VU University Medical Centre Intervention: ICG Comparison: IOC					
Outcomes	Anticipated absolute effects* (95% CI)	Relative effect (95% CI)	No. of participants (studies)	Quality of the evidence [22]	Comments
	Risk with IOC	Risk with ICG			
Cystic duct	Study population	RR 1.16 (1.00–1.35)	430 (four observational studies)	Moderate^a,b,c,d,e^	Downgraded for imprecision
	837 per 1000	971 per 1000 (837–1000)			
Common bile duct	Study population	RR 1.00 (0.97–1.03)	430 (four observational studies)	Moderate^e,f^	Downgraded for imprecision
	851 per 1000	851 per 1000 (826–877)			
Common hepatic duct	Study population	RR 0.76 (0.58–1.01)	300 (three observational studies)	Low^b,e,f,g^	Downgraded for imprecision and serious risk of bias
	793 per 1000	603 per 1000 (460–801)			
*The risk in the intervention group (and its 95% confidence interval) is based on the assumed risk in the comparison group and the relative effect of the intervention (and its 95% CI)					
CI: Confidence interval; RR: risk ratio					
GRADE Working Group grades of evidence					
High quality: we are very confident that the true effect lies close to that of the estimate of the effect					
Moderate quality: we are moderately confident in the effect estimate that the true effect is likely to be close to the estimate of the effect, but there is a possibility that it is substantially different					
Low quality: our confidence in the effect estimate is limited that the true effect may be substantially different from the estimate of the effect					
Very low quality: we have very little confidence in the effect estimate that the true effect is likely to be substantially different from the estimate of effect					

^a^Test for heterogeneity *p* < 0.0001

^b^ *I* ^2^ > 50%

^c^Small variation in size effect

^d^Overlapping of CI

^e^Two studies (Osayi/Prevot) include only patients with uncomplicated gallbladder disease

^f^CI crosses clinical decision threshold

^g^Selection bias

### Obesity and visualization accuracy

In addition to using BMI as a baseline characteristic, three studies reported on ICG visualization in different BMI groups [27, 28, 35]. Osayi et al. [35] reported similar visualization rates for the CD, CBD and CHD, but significantly increased visualization of the CD junction in the BMI ≤ 30 group (*p* = 0.038). Analysis of 20 patients with BMI > 35 revealed prevalence of visualization of the CD and CBD of 91 and 64%. Dip et al. [28] evaluated 71 patients of whom 53% had a BMI > 30. They reported no statistically significant differences between obese (BMI > 30) and non-obese (BMI ≤ 30) patients for biliary tract visualization. Obesity was mentioned as a cause of failure of ICG visualization of bile ducts in one study [15].

Buchs et al. [27] noted a significant faster dissection in the ICG group compared to the control group (conventional laparoscopy) in patients with a BMI < 25. Biliary tract visualization was not assessed.

## Discussion

This systematic review has examined the use of near-infrared imaging using ICG for visualization of the cystic duct and related structures. Results suggest that the use of the near-infrared imaging with ICG technique provides good overall visualization rates of the CD, CBD, CHD and CD junction prior to and following dissection of Calot’s triangle. Although the number of studies is limited and, therefore, the strength of our conclusions is also limited, visualization rates of the biliary structures using near-infrared imaging techniques with ICG appear to be equally good for either 2.5 mg fixed dosage or 0.05 mg per kg dosage of ICG. Small variation of timing of ICG administration was seen, varying between over an hour before surgery until directly after anaesthesia. As of yet, studies have revealed little information regarding influence of obesity or gallbladder disease aetiology on biliary tract visualization. Also, biliary tract visualization was comparable for near-infrared imaging with ICG and conventional IOC.

Most studies used similar timing of ICG administration. Only Kono et al. [33] described in their cohort a difference in visualization of the CD-j after different administration times, with significantly more CD-j visualization after a longer preoperative administration of ICG (median 90 min prior to surgery (range 15–165) versus 47 min (range 21–205), *p* < 0.01). Verbeek et al. [41] described that administration of ICG 24 h prior to surgery results in a significantly increased CBD-to-liver contrast. Longer preoperative administration of ICG could result in increased visualization rates of the biliary tract structures.

An important factor that could influence ICG imaging is the amount of intra-abdominal adipose tissue. Unfortunately, too few studies have examined this aspect and; therefore, strong conclusions cannot be derived. Interestingly, one study [35] reported improved visualization rates for the CD-j in patients with lower BMI, while another study reported no differences [28] with increasing BMI. Intra-abdominal adipose tissue has been known to be a risk factor in abdominal surgery, leading to increased number of post-operative complications [42]. More intra-abdominal adipose tissue results in a decreased penetration of near-infrared light and thus decreased visualization of biliary tract structures [33]. BMI has been used as a standard for obesity because of its simplicity; however, BMI does not distinguish between the type of adipose tissue or location of fat within the intra-abdominal cavity [43, 44]. In order to assess the effects of intra-abdominal adipose tissue on near-infrared imaging with ICG, the patients should be selected on basis of fat percentage assessed by either CT or MRI instead of BMI [45].

There is moderate quality evidence comparing near-infrared imaging using ICG to conventional IOC; therefore, future research is likely to have an important impact on confidence in the estimate of effect and may change the estimate. However, since IOC comes with higher costs, more difficult perioperative logistics, greater radiation exposure, greater use of radiographic contrast fluids, frequent technical failure and risk of bile duct injury due to cannulation of the CD, ICG might be considered to be the better option for visualization of the biliary tract [46], although further research is necessary to confirm this recommendation.

As IOC depends on at least partial dissection in order to cannulate the CD, ICG provides early imaging before start of dissection. Not only are early (prior to dissection) identification rates with ICG adequate, ICG can be used multiple times during dissection without increasing radiation or contrast load to the patient compared to IOC. Also, overall costs for ICG are 32.3 USD compared to 778.83 USD for IOC [46], although cost for the near-infrared imaging system is unclear.

Several studies have reported aberrant biliary anatomy with the use of ICG [16, 30, 34, 40]. Most commonly reported aberrant anatomy are variations in the hepatic duct. Failure to identify aberrant biliary anatomy could result in bile duct injury. In order to reduce major post-operative complications, ICG identification of aberrant anatomy might lead to a reduced rate of biliary tract leakages.

This systematic review is limited by the design of the included studies. Most included studies are prospective cohort studies and, therefore, highly subject to bias. To date, no randomized trials have been performed that investigate biliary tract visualization. The design of these prospective cohort studies is also limited because most studies did not compare intraoperative visualization with ICG to conventional white light laparoscopy. Therefore, no conclusions can be drawn whether the near-infrared with ICG technique provides advantages over conventional laparoscopy.

In addition, a rather heterogeneous population was examined. Some studies included only uncomplicated gallbladder disease, while others have included both complicated and uncomplicated disease. In the studies reporting both indications, biliary tract visualization results for both complicated and uncomplicated are pooled together. Furthermore, studies used different definitions for uncomplicated and complicated gallbladder disease. Therefore, no conclusions can be drawn on the use of biliary tract visualization for either complicated or uncomplicated gallbladder disease.

This systematic review and meta-analysis emphasize the need for further investigation. Randomized trials comparing near-infrared imaging with ICG to conventional white light laparoscopy are needed to assess the additive value of near-infrared imaging with ICG. Also, the effect of earlier preoperative ICG administration on biliary tract visualization should be further evaluated. To analyse the effect of uncomplicated and complicated gallbladder disease on ICG visualization results, further research should clearly define included patients groups.

## Conclusion

This systematic review shows that near-infrared imaging with ICG provides good visualization of the biliary structures during LC. Visualization rates were equal for both dosage schemes. When compared to IOC, ICG provides equal visualization of the bile ducts before dissection. Near-infrared imaging with ICG has the potential to replace IOC for biliary mapping. However, future research is necessary for optimization and standardization of the near-infrared ICG technique.

## Electronic supplementary material

Below is the link to the electronic supplementary material.

Supplementary material 1 (DOCX 20 kb)
