# Supplementary material for: Biliary tract visualization using near-infrared imaging with indocyanine green during laparoscopic cholecystectomy: results of a systematic review
Source: Surg Endosc. 2016 Nov 14;31(7):2731–42. doi: 10.1007/s00464-016-5318-7 (PMC5487840; doi:10.1007/s00464-016-5318-7)
Supplement: Supplementary file 1 — Supplementary material 1 (DOCX 20 kb) [file 464_2016_5318_MOESM1_ESM.docx]

**SUPPLEMENTARY INFORMATION**

1. Search strategy and search results:

**Supplementary information S1**

**Search strategy for PubMed (February 8^th^ 2016)**

[Mesh] = Medical subject headings

[tiab] = words in title or abstract

| **Search** | **Query** | **Items found** |
| --- | --- | --- |
| #4 | #1 AND #2 AND #3 | 244 |
| #3 | "Indocyanine Green"[Mesh] OR "Fluorescent Dyes"[Mesh] OR indocyanine green[tiab] OR wofaverdin[tiab] OR vophaverdin[tiab] OR icg[tiab] OR fluorescen*[tiab] OR fluorochrome*[tiab] OR fluorogenic*[tiab] OR cw800*[tiab] OR irdye*[tiab] OR fluorophore[tiab] OR fluorescein[tiab] OR indocyanine green[ot] OR wofaverdin[ot] OR vophaverdin[ot] OR icg[ot] OR fluorescen*[ot] OR fluorochrome*[ot] OR fluorogenic*[ot] OR cw800*[ot] OR irdye*[ot] OR fluorophore[ot] OR fluorescein[ot] | 405300 |
| #2 | "Cholangiography"[Mesh] OR "Spectroscopy, Near-Infrared"[Mesh] OR "Surgery, Computer-Assisted"[Mesh] OR cholangiogra*[tiab] OR cholangiopancreatograph*[tiab] OR near-infrared[tiab] OR nir[tiab] OR image guided surger*[tiab] OR computer-aided surger*[tiab] OR computer assisted surger*[tiab] OR robot*[tiab] OR fireflight[tiab] OR biliary map*[tiab] OR angiogra*[tiab] OR ((real time[tiab] OR realtime[tiab] OR biliary[tiab] OR optical[tiab] OR intra-operative[tiab] OR intraoperative[tiab]) AND (imag*[tiab] OR visual*[tiab])) OR cholangiogra*[ot] OR cholangiopancreatograph*[ot] OR near-infrared[ot] OR nir[ot] OR image guided surger*[ot] OR computer-aided surger*[ot] OR computer assisted surger*[ot] OR robot*[ot] OR fireflight[ot] OR biliary map*[ot] OR angiogra*[ot] OR ((real time[ot] OR realtime[ot] OR biliary[ot] OR optical[ot] OR intra-operative[ot] OR intraoperative[ot]) AND (imag*[ot] OR visual*[ot])) | 321028 |
| #1 | "Biliary Tract Surgical Procedures"[Mesh] OR "Biliary Tract"[Mesh] OR "Biliary Tract Diseases"[Mesh] OR Biliar*[tiab] OR gallbladder[tiab] OR bile duct*[tiab] OR ampulla of vater*[tiab] OR cystic duct*[tiab] OR hepatic duct*[tiab] OR cholecystectom*[tiab] OR choledochostom*[tiab] OR cholelithiasis[tiab] OR cholangitis[tiab] OR choledoch*[tiab] OR cholecyst*[tiab] OR gallstone*[tiab] OR hepatobiliary*[tiab] OR gall bladder[tiab] OR biliar*[ot] OR gallbladder[ot] OR bile duct*[ot] OR ampulla of vater*[ot] OR cystic duct*[ot] OR hepatic duct*[ot] OR cholecystectom*[ot] OR choledochostom*[ot] OR cholelithiasis[ot] OR cholangitis[ot] OR choledoch*[ot] OR cholecyst*[ot] OR gallstone*[ot] OR hepatobiliary*[ot] OR gall bladder[ot] | 207453 |

1. **Search strategy for Embase.com (February 8^th^ 2016)**

/exp = EMtree keyword with explosion

:ab,ti = words in title or abstract

NEAR/3= words near to each other, 3 places apart

NEXT/1= words next to each other, 1 place apart

| **Search** | **Query** | **Items found** |
| --- | --- | --- |
| #4 | #1 AND #2 AND #3 | 1227 |
| #3 | 'indocyanine green'/exp OR 'fluorescent dye'/exp OR 'indocyanine green':ab,ti OR wofaverdin:ab,ti OR vophaverdin:ab,ti OR icg:ab,ti OR fluorescen*:ab,ti OR fluorochrome*:ab,ti OR fluorogenic*:ab,ti OR cw800*:ab,ti OR irdye*:ab,ti OR fluorescein:ab,ti | 505845 |
| #2 | 'cholangiography'/exp OR 'near infrared spectroscopy'/exp OR 'computer assisted surgery'/exp OR cholangiogra*:ab,ti OR cholangiopancreatograph*:ab,ti OR 'near infrared':ab,ti OR nir:ab,ti OR 'image guided surgery':ab,ti OR 'image guided surgeries':ab,ti OR ('real time' NEAR/3 imag*):ab,ti OR (realtime NEAR/3 imag*):ab,ti OR (optical NEAR/3 imag*):ab,ti OR ('computer aided' NEAR/3 surger*):ab,ti OR ('computer assisted' NEAR/3 surger*):ab,ti OR robot*:ab,ti OR fireflight:ab,ti OR (biliary NEAR/3 map*):ab,ti OR visual*:ab,ti | 643692 |
| #1 | 'biliary tract surgery'/exp OR 'hepatobiliary system'/exp OR 'biliary tract disease'/exp OR biliar*:ab,ti OR gallbladder:ab,ti OR (bile NEXT/1 duct*):ab,ti OR 'ampulla of vater':ab,ti OR (cystic NEXT/1 duct*):ab,ti OR (hepatic NEXT/1 duct*):ab,ti OR cholecystectom*:ab,ti OR choledochostom*:ab,ti OR cholelithiasis:ab,ti OR cholangitis:ab,ti OR choledoch*:ab,ti OR cholecyst*:ab,ti OR gallstone*:ab,ti OR hepatobiliary*:ab,ti OR 'gall bladder':ab,ti | 793399 |

1. **Search strategy for Wiley/Cochrane Library (February 8^th^ 2016)**

ti,ab,kw = words in title, abstract or keyword

| **Search** | **Query** | **Items found** |
| --- | --- | --- |
| #1 | biliar* or gallbladder or (bile and duct*) or (ampulla and vater*) or (cystic and duct*) or (hepatic and duct*) or cholecystectom* or choledochostom* or cholelithiasis or cholangitis or choledoch* or cholecyst* or gallstone* or hepatobiliary* or gall bladder | 9099 |
| #2 | cholangiogra* or cholangiopancreatograph* or near-infrared or nir or (image guided and surger*) or (computer-aided and surger*) or (computer assisted and surger*) or robot* or fireflight or (biliary and map*) or angiogra* or ((real time or realtime or biliary or optical or intra-operative or intraoperative) and (imag* or visual*)) | 22320 |
| #3 | indocyanine green or wofaverdin or vophaverdin or icg or fluorescen* or fluorochrome* or fluorogenic* or cw800* or irdye* or fluorophore or fluorescein | 4684 |
| #4 | #1 and #2 and #3 | 20 |

1. **Search strategy for Web of Science (February 8^th^ 2016)**

TS = words in topic

TI = words in title

| **Search** | **Query** | **Items found** |
| --- | --- | --- |
| #4 | #3 AND #2 AND #1 | 390 |
| #3 | TS=(‘indocyanine green’ OR wofaverdin OR vophaverdin OR icg OR fluorescen* OR fluorochrome* OR fluorogenic* OR cw800* OR irdye* OR fluorophore OR fluorescein) | 1,163,990 |
| #2 | TS=(cholangiogra* OR cholangiopancreatograph* OR 'near-infrared' OR 'near infrared' OR nir OR (image guided AND surger*) OR (computer-aided AND surger*) OR (computer assisted AND surger*) OR robot* OR fireflight OR (biliary AND map*) OR angiogra* OR ((real time OR realtime OR biliary OR optical OR 'intra-operative' OR intraoperative) AND (imag* OR visual*))) | 943,839 |
| #1 | TS=(biliar* OR gallbladder OR (bile AND duct*) OR (ampulla AND vater*) OR (cystic AND duct*) OR (hepatic AND duct*) OR cholecystectom* OR choledochostom* OR cholelithiasis OR cholangitis OR choledoch* OR cholecyst* OR gallstone* OR hepatobiliary* OR ‘gall bladder’) | 370,644 |
